# Supplementary material for: Relationship of serum copper and HLADR4 tissue typing to disease activity and severity in patients with rheumatoid arthritis: A cross sectional study
Source: Ann Med Surg (Lond). 2021 Dec 24;73:103193. doi: 10.1016/j.amsu.2021.103193 (PMC8767240; doi:10.1016/j.amsu.2021.103193)
Supplement: Multimedia component 1 [file mmc1.doc]

| **The STROCSS 2019 Guideline** | | |
| --- | --- | --- |
| **Item no.** | **Item description** | **Page** |
| **TITLE** | | |
| 1 | Title: Relationship of Serum copper and HLADR4 tissue typing to disease activity and severity in patients with rheumatoid arthritis: A cross sectional study   - Key elements of study design are stated: prospective cross sectional study | 1  1, 4 |
| **ABSTRACT** | | |
| 2a | **Background:** Rheumatoid arthritis is a chronic disabling disease associated with high burden on individuals and national health system. Early detection of rheumatoid arthritis is helpful in management and preventing further complications. | 1 |
| 2b | **Methods:** This study was an observational prospective cross sectional study implemented in the Rzgary general teaching hospital in Erbil governorate-Iraq from 1^st^ of November 2019, to 31^st^ of October 2020. Study sample included fifty rheumatoid arthritis patients. The diagnosis of rheumatoid arthritis was done according to 2010-American College of Rheumatology-European League against Rheumatism criteria. The serum copper and HLA-DR4 typing were assessed in regard to activity and severity of rheumatoid arthritis patients. | 1 |
| 2c | **Results:** This study found that high serum copper level was directly related to both active and severe rheumatoid arthritis disease (p<0.001). Additionally, this study revealed that positive HLADR4 typing was also related to both active and severe rheumatoid arthritis disease (p<0.001). This study revealed no significant relationship between rheumatoid arthritis activity and severity with HLADR4 subtypes. | 1 |
| 2d | **Conclusions:** The serum copper level and HLA-DR typing of patients with rheumatoid arthritis may be useful in prediction of rheumatoid arthritis disease activity and severity. | 1 |
| **INTRODUCTION** | | |
| 3 | **Introduction:** Rheumatoid arthritis (RA) is a chronic systemic inflammatory autoimmune disease affecting originally the joints and finally multiple body organs. It primarily destroys the bones and cartilages causing damage to tendons and ligaments. Early RA is presented with symptoms in less than six months duration, while established RA is presented with symptoms in six months duration and more ^1-3^. The morning joints stiffness, fever, fatigue, weight loss, tender, warm swollen joints with rheumatoid nodules are the predominant symptoms of RA. The RA is an adulthood disease but it also affects children (juvenile RA) ^4, 5^. The global prevalence of RA is 1% ^6^; in Western countries it is ranged between 1-2% ^7^ and in Eastern Mediterranean countries is 0.62% ^8^.  The exact pathogenesis of RA is still unknown. However, etiology of RA might be the outcome of both genetic and environmental effects. The risk of RA is affected by Human Leukocyte Antigen (HLA)-DRB1 gene alleles (HLA-DRB1*04, HLA-DRB1*01 and HLA-DRB1*10) these alleles are used as markers for severity of RA^9^. The epigenetics (evidence of hereditary effect with no changes in DNA sequence) is shown to play role in development of RA ^10^. Cigarette smoking is considered as the main environmental risk factor related to RA in addition to the effect of intestinal microbiome changes ^11, 12^. In regard to anti-citrullinated protein antibodies (ACPAs), the RA is classified into two subtypes, which are different in pathophysiology, diagnosis and treatment ^13, 14^. The pathogenesis of RA involves four stages (triggering, maturation, targeting and fulminant) ^15^.  The diagnosis of RA is depending mainly on clinical symptoms, physical examination, family history, laboratory and imaging results (erythrocyte sedimentation rate {ESR}, RA factor, ACPA, C-reactive protein {CRP}, x-ray, ultrasound and magnetic resonance imaging ^16, 17^. Diagnosis of RA is focusing on differentiating it from osteoarthritis ^18, 19^. The 2010 ACR-EULAR (American College of Rheumatology-European League against Rheumatism) criteria included joints involvement (0-5), serology (0-3), acute phase reactants (0-1) and symptoms duration (0-1), when the total score is more than 6 and other causes of synovitis is excluded, the RA diagnosis is proved ^20^. The RA treatment aimed to decrease joint inflammation, lowering the pain, increasing joint function and inhibiting the joint damage and deformity. The RA treatment involves pharmaceuticals (nonsteroidal anti-inflammatory drugs {NSAIDs}, disease-modifying antirheumatic drugs {DMARDs} and new biological agents), weight-bearing exercise, medical education and rest ^21^.  RA disease progression is variable from remission cases to rapidly progressive and severe cases. Assessment of RA disease activity and severity is helpful in early diagnosis, better planning of treatment and preventing future complications ^22^. Available RA disease activity scores are simplified disease activity index (SDAI), clinical disease activity index (CDAI), disease activity Score 28-joint count (DAS28)-ESR and DAS-CRP. The DAS28 score are ranged between 0-9.4 and assumed by tender joints, swollen joints, general health and inflammatory markers ^23^. The DAS28 could use the ESR or CRP levels ^24^. The DAS28-ESR is used as a measure of RA disease activity ^25^, determinant of biological treatment ^26^, RA classification in clinical trials ^23^, therapy outcome target ^27^ and predictor of RA severity (>5.1) ^28^.  The inflammatory and biochemical markers are important in either predicting clinical response or predicting and monitoring the joints damage ^29^. The copper (Cu) is a significant chemical constituent needed by normal human body for growing and developing. The Cu is required for maturating collagen tissues. Different literatures showed the Cu participation in pathogenesis of RA ^30-32^.  The prevalence of RA among Iraqi population was (1%) in previous century, ^33^ that increased gradually to 1.6% at 2001, and 3.02% at 2011 with epidemiological transition of Iraqi community in last twenty years ^34^. Iraqi RA patients are characterized by high activity and severity of the disease with low quality of life related to low national health infrastructure and low educational and socioeconomic status of patients in addition to scarcity of researches on improving the early identification and diagnosis of RA ^35^. For all of these reasons, this study aimed to assess the relationship between serum copper and RA activity with identifying the relation between HLA-DR tissue typing and RA severity. | 2-3 |
| **METHODS** | | |
| 4a | Registration and ethics The study ethics were implemented in regard to Helsinki Declaration by documented agreement of patients, approved by ethical committee in Hawler Medical University and management of RA patients accordingly. The methods of this article were prepared according to STROCSS criteria ^36^. The research was registered at research registry: [https://www.researchregistry.com](https://www.researchregistry.com/). With unique identifying number researchregistry 7181. Samples of fifty RA patients were enrolled in this study. | 4,17 |
| 4b | Ethical Approval: The study ethics were implemented in regard to Helsinki Declaration by documented agreement of patients, approved by ethical committee in Hawler Medical University and management of RA patients accordingly. | 4,17 |
| 4c | Protocol: This study is an observational prospective cross sectional study implemented in the Rzgary General Teaching Hospital in Erbil governorate-Iraq from 1^st^ of November 2019, to 31^st^ of October 2020. The studied sample was all RA patients attended to Rheumatology Consultancy clinic of Rzgary hospital. Inclusion criteria were adult patients with rheumatoid arthritis in remission or active disease, diagnosed and classified according to the 2010/ACR-EULAR characteristics depending on DAS28-(ESR) score | 4 |
| 4d | Patient Involvement in Research  The studied sample was all RA patients attended to Rheumatology Consultancy clinic of Rzgary hospital. Inclusion criteria were adult patients with rheumatoid arthritis in remission or active disease, diagnosed and classified according to the 2010/ACR-EULAR characteristics depending on DAS28-(ESR) score | 4 |
| 5a | Study Design: prospective cross sectional study | 4 |
| 5b | This study is an observational prospective cross sectional study implemented in the Rzgary General Teaching Hospital in Erbil governorate-Iraq from 1^st^ of November 2019, to 31^st^ of October 2020. The studied sample was all RA patients attended to Rheumatology Consultancy clinic of Rzgary hospital. Inclusion criteria were adult patients with rheumatoid arthritis in remission or active disease, diagnosed and classified according to the 2010/ACR-EULAR characteristics depending on DAS28-(ESR) score ^20^. Exclusion criteria were younger age, other acute inflammatory diseases, kidney and liver disorders, infections of urinary and genital tracts, Hansen's disease, cancer, pregnancy, females using oral contraceptive pills, use of copper utensils for cooking and feeding and patients refused to be enrolled in the study. The study ethics were implemented in regard to Helsinki Declaration by documented agreement of patients, approved by ethical committee in Hawler Medical University and management of RA patients accordingly. The methods of this article were prepared according to STROCSS criteria ^36^. The research was registered at research registry: [https://www.researchregistry.com](https://www.researchregistry.com/). With unique identifying number researchregistry 7181. Samples of fifty RA patients were enrolled in this study.  Information of patients was collected directly by researcher through a prepared questionnaire designed by the researcher according to previous literatures ^30-32^. The questionnaire included general characteristics of RA patients (age, gender, race, body mass index, previous copper wearing and RA disease duration), RA disease characteristics (rheumatoid factor, ACPA, rheumatoid nodule, x-ray of hand with bone erosions, morning stiffness, ESR level, Hb level, disease activity and severity according to DAS28 score), serum copper and HLADR4 types and subtypes of RA patients. Remitted RA (DAS<2.6), while activity (DAS28 from 2.6 and more) was considered as active disease ^37^. The severity of RA patients was categorized according to American college of Rheumatology (ACR) features of disease activity score (DAS 28) into: patient in remission (DAS<2.6), patients with low (DAS28from 2.6 to < 3.2), patient with moderate (DAS28 from 3.2-5.1) and patient with high (DAS28 > 5.1). Measurement of disease activity-28 score was done in regard to erythrocyte sedimentation rate and assessment of Nijmegen formula ^38^. RA disease severity involves activity of RA disease, damage of joints and disabled function, while our study measured RA severity according to DAS28 score ^39^.  4  After taking full history and examination, RA patients were sent for complete blood count, ESR and C-reactive protein (CRP). Anti-CCP was measured by enzyme-linked immunosorbent assay (ELISA) and rheumatoid factor (RF) by the agglutination method. The serum copper level of RA patients was assessed through collecting samples of blood from fasting patients and using of colorimetric laboratory technique within two to three hours of samples collection. X-rays of hands and wrist (postero-anterior) views are taken for all patients, and its report written by an experienced radiologist to detect any bone erosion. The HLA-DR typing was acquired through DNA analysis (PCR-RFLP) technique and the subtypes of HLADR were determined by the PCR-RFLP technique for all studied patients.  The RA patients' information was interpreted statistically by SPSS program-26. Chi square and Fishers exact tests were implemented, and p value of ≤0.05 considered as significant. | 4-5 |
| **RESULTS** | | |
|  | This study included fifty rheumatoid arthritis patients. The mean age of patients was 50.04 years and predominant age category was (50-59 years). Female gender was more than male gender with female: male ratio (2.5:1). The residence of RA patients was urban for 52% of them and rural for 48% of them. The race of RA patients was Kurdish for half of them and Arabic for the other half. About one third (30%) of RA patients were overweight and the previous wearing of copper was detected in 12% of them. More than half (58%) of studied patients had disease duration of ten years and more. (***Table 1***)  The rheumatoid factor was positive in 76% patients, while the anticetrulinated peptide antibody was positive among 68% patients and the rheumatoid nodule was positive among 22% patients. X-ray of hand with bone erosions was positive in 54% of RA patients and the morning stiffness was positive in 64% of them. Hemoglobin level of RA patients was normal in 42% of them and low in 58% of them. The Erythrocyte sedimentation rate (ESR) level was normal in 18% of RA patients, while mild in 56% of them, moderate in 14% of them and high in 12% of them. The disease activity score 28 was less than 2.6 in 48% of them and 2.6 and more in 52% of them. Severity of RA according to DAS28 revealed that remission of RA was present in 46% of patients, while active RA disease was present in 54% of them. RA disease severity classification according DAS28 showed that 18% of patients had low activity, 34% of them had moderate activity and only one patient had high activity. (***Table 2***)  Mean serum copper level of RA patients was (147.2 mg/dl); 54% of RA patients had high serum copper level. The HLADR4 typing of RA patients was positive in 60% of them; the HLADR4 subtypes were 404 (63.3%), 405 (20%) and 411 (16.7%). (***Table 3***)  RA patients' features like age, gender, residence and race were not significantly related to DAS-28 of RA disease. Overweight patients were significantly related to active RA disease (p=0.01). Previous copper wearing and long RA duration were significantly related to active RA disease (p=0.01, p<0.001, respectively). (***Table 4***)  The Rheumatoid factor was not significantly related to RA disease activity (p=0.7). Positive anticetrulinated peptide antibody, RA nodule, **X**-ray of hand with bone erosions and morning stiffness were significantly related to active RA disease (p<0.001). The anemic patients were significantly related to active RA disease (p<0.001). High ESR level was significantly related to active RA disease (p<0.001). (***Table 5***)  There was a highly significant association between high serum copper level and active RA disease (p<0.001). Positive HLADR4 typing was significantly related to active RA disease (p<0.001). HLADR4 subtypes of RA patients were not significantly related to disease activity (p=0.2). (***Table 6***)  There was a highly significant association between high serum copper level and moderate severity of RA (p<0.001). HLADR4 typing was significantly related to severe RA disease (p<0.001). HLADR4 subtypes of RA patients were not significantly related to disease severity (p=0.4). (***Table 7***) | 5-13 |
| 10b | Participant Comparison: the following areas are described comprehensively   - Table comparing demographics included - Differences, with statistical relevance - Any group matching, with methods | 5-13 |
| 10c | Intervention: the following areas are described comprehensively   - Changes to interventions, with rationale and diagram, if appropriate - Learning required for interventions - Degree of novelty for intervention | 5-13 |
| 11a | Outcomes: the following areas are described comprehensively   - Clinician-assessed and patient-reported outcomes for each group - Relevant photographs and imaging are desirable - Confounders to outcomes and which are adjusted | 5-13 |
| 11b | Tolerance: the following areas are described comprehensively   - Assessment of tolerance - Loss to follow up, with reasons (percentage and fraction) - Cross-over with explanation | 5-13 |
| 11c | Complications: the following areas are described comprehensively   - Adverse events described - Classified according to Clavien-Dindo classification* - Mitigation for adverse events (blood loss, wound care, revision surgery should be specified)   *Dindo D, Demartines N, Clavien P-A. Classification of Surgical Complications. A New Proposal with Evaluation in a Cohort of 6336 Patients and Results of a Survey. Ann Surg. 2004; 240(2): 205-213 | 10-12 |
| 12 | Key Results: the following areas are described comprehensively   - Key results, including relevant raw data - Statistical analyses with significance | 5-13 |
| **DISCUSSION** | | |
| 13 | Discussion: Rheumatoid arthritis is a chronic disease with variable course and outcomes. Prognostic markers of rheumatoid arthritis are essential in prediction of diseases activity, spectrum of joints damage, functional status and disease severity, which are important in developing management and rehabilitations plans and avoiding complications ^40^.  Present study found mean serum copper in RA patients was (147.2 mg/dl), 54% of RA patients had high serum copper. These results are close to outcomes of Xin et al ^29^ meta-analysis study in China that found high proportion of RA patients with high serum copper and low serum zinc levels. Our study found a high serum copper level relation with active RA disease. Consistently, Chakraporty et al ^41^ study in India who reported that serum copper level might be used as predictor of rheumatoid arthritis activity. Strecker et al ^31^ found that high serum copper level among active rheumatoid arthritis patients is correlated to the inflammatory process. Inconsistently, Moustafa et al ^32^ case control study in Iraq revealed low serum level of copper in RA patients in comparison to normal individuals. This inconsistency might be due to the fact that previous Iraqi study neglected the diseases activity and its relationship with serum copper. The current study found a highly significant association between high serum copper level and moderate severity of RA. Parallel to our results, Yang et al ^42^ study in China who detected high serum copper level in active RA patients in comparison to normal people. The Cu is important trace element for human body required for metabolism of different enzymes as it plays roles in electrons receiving and donation in physiological human processes. The Cu ions also play a significant role in stress induced non-classical export of fibroblast growth factor-1 (FGF-1) and interleukin-1α (IL-1α) that play roles in activity and severity of RA ^43^. It was shown that serum Cu level in rheumatoid arthritis patients was elevated ^44^ and the Cu level in RA patients was positively correlated to IL-1β and tumor necrosis factor-α (TNF-α) ^45^. This high serum copper level among RA patients could be used in adding copper as early biomarker in confirming diagnosis of RA and assessing activity of diseases which in turn helping for planning of RA management and monitoring in addition to prognostic importance. Our study revealed (145 mg/dl) as an appropriate cutoff value of serum Cu in prediction of RA activity with acceptable validity measures (sensitivity 96.3%, specificity 95.8% and accuracy 95%). Similarly, Gosselt et al ^46^ study in Netherlands recommended importance of identifying new biomarkers predicting disease activity of RA which help in diagnosis and monitoring.  Current study showed that HLA-DR4 typing of RA patients was positive in 60% of them; the HLADR4 subtypes were 404 (63.3%), 405 (20%) and 411 (16.7%). These results are close to findings of Salesi et al ^47^ project in Iran who reported that 35% of RA patients had positive HLA-DR4 and the main subtype was HLADR4-404. In Iraq, a study conducted by Al-Yasiri et al ^48^ stated that HLA-DRB1*04 genotype contributed significantly to development of rheumatoid arthritis. This difference might be due to discrepancy in laboratory findings and methodology between studies. Our study showed a highly significant association observed between positive HLADR4 typing and active RA disease. This result coincides with reports of Drongelen and Holoshitz study in USA ^49^. Another Iraqi study carried out in Erbil city by Al-Timimi et al ^50^ found that HLA-DQB1*06 was the common genotype of RA patients that was related to RA disease activity. This inconsistency might be due to differences in laboratory techniques and inclusion criteria of the studies. The present study found that positive HLADR4 typing was related to severe RA disease. This result is consistent with findings of different studies like Mahdi study in Iraq ^51^ and Larid et al ^52^ study in France which reported a strong link between HLA-DR4 typing and severe rheumatoid arthritis disease. The RA disease is resulted from combination of genetic and environmental factors with risk of severe disease in patients with specific genetic types ^53^. However, the present study showed no significant relationship between HLA-DR4 alleles and RA disease activity or severity. In same direction, Almeida et al ^54^ study in Brazil found no statistically significant relationship between HLA-DR genetic subtypes and RA disease activity.  In present study, overweight patients were related to active RA. This result is consistent with findings of Gremese et al ^55^ study in Italy. Our study revealed a significant association between previous copper wearing and active RA disease. This result is similar to findings of Richmond et al ^56^ study in United Kingdom. In our study, longer RA disease duration was related to active disease. Similarly, Aletaha et al ^57^ study in Austria revealed that longer RA disease duration is related to active disease. Positive anticetrulinated peptide antibody, RA nodule, **x**-ray of hand with bone erosions, morning stiffness high ESR in present study were significantly related to active RA disease (p<0.001). These results are similar to different studies ^13, 16, 20^. Current study reported that anemia was related to active RA disease. Consistently, Goyal et al ^58^ study in India documented that anemia is prevalent in RA patients and the hemoglobin level was negatively correlated to RA disease activity. Our study showed that both copper wearing and RA disease duration significantly affecting the serum copper level in studied patients. However, the number of affected patients with copper wearing was only six patients that didn’t affect the results of the study, and the RA duration is related to RA disease and not considered as confounding variable.  The novel findings in the present study are the relationship of serum copper level with activity of RA disease and relation of HLA-DR4 tissue typing with RA severity that could be helpful in diagnosis and management of RA disease. Limitations of the present study were those related to cross sectional study design (inability to assess temporal relationship), single center study and small sample size related to the fact that high proportion of RA patients preferred private clinic on governmental health centers in addition to exclusion criteria related to copper confounding variables.  In conclusion, the serum copper level and HLA-DR typing of patients with rheumatoid arthritis are related to rheumatoid arthritis disease activity. The serum copper level of RA patients is significant predictor of active disease, while the HLA-DR4 typing is significant predictor of severe RA disease. This study recommends the importance of serum copper assessment, which might be useful in RA diagnosing and monitoring with implementing HLA tissue typing in assessing severity of RA disease. Further national longitudinal studies on role of serum copper in diagnosis and staging of RA disease should be supported. | 14-16 |
| 14 | Strengths and Limitations:  Limitations of the present study were those related to cross sectional study design (inability to assess temporal relationship), single center study and small sample size related to the fact that high proportion of RA patients preferred private clinic on governmental health centers in addition to exclusion criteria related to copper confounding variables. | 16 |
| **CONCLUSION** | | |
| 16 | Conclusions:  In conclusion, the serum copper level and HLA-DR typing of patients with rheumatoid arthritis are related to rheumatoid arthritis disease activity. The serum copper level of RA patients is significant predictor of active disease, while the HLA-DR4 typing is significant predictor of severe RA disease. This study recommends the importance of serum copper assessment, which might be useful in RA diagnosing and monitoring with implementing HLA tissue typing in assessing severity of RA disease. Further national longitudinal studies on role of serum copper in diagnosis and staging of RA disease should be supported. | 16 |
| **DECLARATIONS** | | |
| 17a | Conflicts of interest  None. | 16 |
| 17b | Funding  No funding | 16 |
