# Supplementary material for: Relationship of serum copper and HLADR4 tissue typing to disease activity and severity in patients with rheumatoid arthritis: A cross sectional study
Source: Ann Med Surg (Lond). 2021 Dec 24;73:103193. doi: 10.1016/j.amsu.2021.103193 (PMC8767240; doi:10.1016/j.amsu.2021.103193)
Supplement: Multimedia component 2 [file mmc2.doc]

STROCSS Checklist

Relationship of Serum copper and HLADR4 tissue typing to disease activity and severity in patients with rheumatoid arthritis

| **Outline** | **Page number** |
| --- | --- |
| 1. Highlights | Sent as separate folder |
| **2. Abstract** | (1) |
| **3.** Keywords | (1) |
| **4. Introduction** | (2-3) |
| **5.  Patients and Methods** | (4-5) |
| **6. Participant selection** | (4) |
| **7.** Statistical analyses | (5) |
| **8. Results** | (5-13) |
| **9.** Discussion | (14-16) |
| **10. Conclusion** | (16) |
| **11.** Ethical approval | (4,17) |
| **12.** Funding | (16) |
| **13.** Conflict of interest | (16) |
| **14.** Research registration unique identifying number | (4,17) |
| **15.** Guarantor | (17) |
| **16.** STROCSS group participants | (4)  **-** Reference number [36], page (21) |
| **17.** References | (18-24) |
